# REJECTION OF CARE, AGGRESSION, AND AGITATION IN PERSONS WITH DEMENTIA: A REVIEW OF DEFINITIONS IN DEMENTIA MEASURES

**DOI:** 10.1093/geroni/igz038.1746

**Published:** 2019-11-08

**Authors:** Scott Choi, Maan Cajita, Laura N Gitlin

**Affiliations:** 1 Towson University, Towson, Maryland, United States; 2 University of Pittsburgh, Pittsburgh, Pennsylvania, United States; 3 Drexel University, Philadephia, Pennsylvania, United States

## Abstract

Objective: To provide a systematic review of how rejection of care, aggression, and agitation are described and operationalized in existing measures of dementia-related behaviors with a particular focus on whether these behaviors are conceptualized as separate phenomena in rating scales. Methods: We reviewed two systematic reviews of behavioral measures in dementia to evaluate their definitions and operationalization of rejection, aggression, and agitation. Additionally, we conducted a systematic review of English-language peer-reviewed articles published from 1980 to 2017 to update the previous list instruments and identify additional measures that were not captured in previous reviews. Results: 43 instruments (23 general behavior measures, 20 symptom-specific measures) developed to measure behavioral symptoms were included. Of these, 25 (58.1%) included items related to rejection of care; 32 (74.4%) included aggression items; and 35 (81.4%) had agitation items. Descriptions and definitions of the behaviors were highly variable across instruments. 13 of 23 general measures and 3 of 20 symptom specific measures included items separately representing all three behaviors while the rest of the measures lacked items measuring one or two behaviors of our interest. Conclusions: The review demonstrated that rejection, aggression, and agitation are measured in most scales yet their measurement is highly variable and they are often not distinguished from each other. Researchers and clinicians need to consider each symptom in its own right and revise existing instruments to address possible misnomers to improve measurement of dementia behaviors.

